# Segment length in cine (SLICE) strain analysis: a practical approach to estimate potential benefit from cardiac resynchronization therapy

**DOI:** 10.1186/s12968-020-00701-4

**Published:** 2021-01-11

**Authors:** Alwin Zweerink, Robin Nijveldt, Natalia J. Braams, Alexander H. Maass, Kevin Vernooy, Frederik J. de Lange, Mathias Meine, Bastiaan Geelhoed, Michiel Rienstra, Isabelle C. van Gelder, Marc A. Vos, Albert C. van Rossum, Cornelis P. Allaart

**Affiliations:** 1 Department of Cardiology, Amsterdam Cardiovascular Sciences (ACS), Amsterdam University Medical Centers (AUMC), Location VU University Medical Center, De Boelelaan 1118, 1081 HV Amsterdam, The Netherlands; 2 Department of Cardiology, Radboud University Medical Center, Nijmegen, The Netherlands; 3 Department of Cardiology, Thoraxcentre, University of Groningen, University Medical Centre Groningen, Groningen, The Netherlands; 4 Department of Cardiology, Maastricht University Medical Centre, Maastricht, The Netherlands; 5 Department of Cardiology, Amsterdam University Medical Centers (AUMC), Location Academic Medical Center, Amsterdam, The Netherlands; 6 Department of Cardiology, University Medical Centre Utrecht, Utrecht, The Netherlands; 7 Department of Medical Physiology, University of Utrecht, Utrecht, The Netherlands

**Keywords:** Cardiovascular magnetic resonance (CMR), Segment length in cine (SLICE), Myocardial strain, Cardiac resynchronization therapy (CRT)

## Abstract

**Background:**

Segment length in cine (SLICE) strain analysis on standard cardiovascular magnetic resonance (CMR) cine images was recently validated against gold standard myocardial tagging. The present study aims to explore predictive value of SLICE for cardiac resynchronization therapy (CRT) response.

**Methods and results:**

Fifty-seven patients with heart failure and left bundle branch block (LBBB) were prospectively enrolled in this multi-center study and underwent CMR examination before CRT implantation. Circumferential strains of the septal and lateral wall were measured by SLICE on short-axis cine images. In addition, timing and strain pattern parameters were assessed. After twelve months, CRT response was quantified by the echocardiographic change in left ventricular (LV) end-systolic volume (LVESV). In contrast to timing parameters, strain pattern parameters being systolic rebound stretch of the septum (SRS_sep_), systolic stretch index (SSI_sep-lat_), and internal stretch factor (ISF_sep-lat_) all correlated significantly with LVESV change (R − 0.56; R − 0.53; and R − 0.58, respectively). Of all strain parameters, end-systolic septal strain (ESS_sep_) showed strongest correlation with LVESV change (R − 0.63). Multivariable analysis showed ESS_sep_ to be independently related to LVESV change together with age and QRS_AREA_.

**Conclusion:**

The practicable SLICE strain technique may help the clinician to estimate potential benefit from CRT by analyzing standard CMR cine images without the need for commercial software. Of all strain parameters, end-systolic septal strain (ESS_sep_) demonstrates the strongest correlation with reverse remodeling after CRT. This parameter may be of special interest in patients with non-strict LBBB morphology for whom CRT benefit is doubted.

## Introduction

Cardiac resynchronization therapy (CRT) is an established therapy for patients with chronic heart failure, reduced left ventricular (LV) ejection fraction and left bundle branch block (LBBB). Patient selection is primarily guided by the electrocardiogram (ECG), but this results in approximately one-third of patients not benefitting from the therapy [1, 2]. Additional criteria are therefore needed to improve patient selection for CRT. Myocardial strain imaging provides mechanical information on LV regional timing differences (also known as dyssynchrony) and inefficient contraction patterns (also known as discoordination). Strain parameters have been linked to CRT outcome in prior studies [3–6]. At present, multiple strain imaging tools are commercially available for both cardiovascular magnetic resonance (CMR) and echocardiographic modalities [7, 8]. CMR strain analysis requires additional tagging sequences (CMR-TAG), or can be performed on standard cine images using specialized feature tracking (CMR-FT) software. In addition, we recently validated the novel segment length in cine (SLICE) technique. The purpose of SLICE is to provide the clinician a simple and transparent method to estimate benefit from CRT. More specifically, SLICE consists of a series of manual frame-to-frame segment length measurements between anatomic landmarks on standard short-axis CMR cines [9]. This technique showed excellent intra-observer and good inter-observer reproducibility for measuring circumferential strains. Moreover, SLICE showed good agreement with gold standard CMR-TAG and holds the advantage of being widely available as it requires no additional image acquisition sequences (i.e. tagging), or commercial post-processing software (i.e. feature tracking). The aim of the present study was to evaluate SLICE as a practicable strain technique to estimate potential benefit from CRT using standard CMR cine images.

## Methods

### Study population

This study was part of the Markers And Response to CRT (MARC) study, a non-randomized, multi-center study that investigated the role of various clinical-, ECG-, echocardiographic-, and biomarker parameters to predict LV reverse remodeling after CRT [10]. The MARC study prospectively enrolled two-hundred-forty patients planned for CRT implantation in six medical centers in the Netherlands. In the present study, 57 patients from five participating centers were included who underwent CMR examination in three of the following experienced centers (Maastricht University Medical Centre, University Medical Centre Groningen and Amsterdam University Medical Centers, location VU medical center; the latter also performing CMR studies for two other participating centers). Previously, we validated SLICE against CMR-TAG in 27 patients who underwent CMR examination with additional CMR-TAG imaging in our center [9]. All subjects gave written informed consent and the local medical ethics committee (Amsterdam University Medical Centers, location VU medical center) approved data collection and management. The investigation conforms with the principles outlined in the Declaration of Helsinki.

### Definition of CRT response

Device programming was DDD mode in all patients with a sensed atrioventricular (AV) delay of 90 ms, paced AV-delay of 130 ms and interventricular (VV) delay 0 ms. Echocardiographic assessment of LV volumes was performed before, and twelve months after CRT implantation. LV end-systolic volume (LVESV) was measured using the biplane Simpson’s method by two experienced observers. Patients with ≥ 15% reduction in LVESV were classified as CRT responders [11].

### Image acquisition

Patients underwent CMR examination using a 1.5 T system (Magnetom Avanto or Aera, Siemens Healthineers, Erlangen, Germany; or Intera CV, Philips Healthcare, Best, The Netherlands). Standard CMR cine images were acquired using a retrospectively ECG-gated balanced steady-state free-precession (bSSFP) sequence with standard short-axis and long-axis orientations to measure LV volumes and calculate LV ejection fraction (LVEF). Typically, temporal resolution was < 50 ms and the number of reconstructed temporal phases within the cardiac cycle was set between 20 and 30. Typical image acquisition parameters were: slice thickness/slice gap: 5/5 mm, 8/0 mm or 6/4 mm; echo time (TE)/repetition time (TR): 1.6 ms/3.2 ms; in-plane spatial resolution: 1.5 by 2.1 mm; flip angle: 45 to 75 degrees. Cine imaging of the LV in the three-chamber view was performed to assess aortic valve closure (AVC). Myocardial scar territory was assessed by late gadolinium enhancement (LGE) imaging, and infarct size was measured using the full width at half maximum method [12]. All CMR data were analyzed using dedicated offline software (QMassMR version 7.6, Medis, Leiden, The Netherlands).

### SLICE strain analysis

**Fig. 1:**
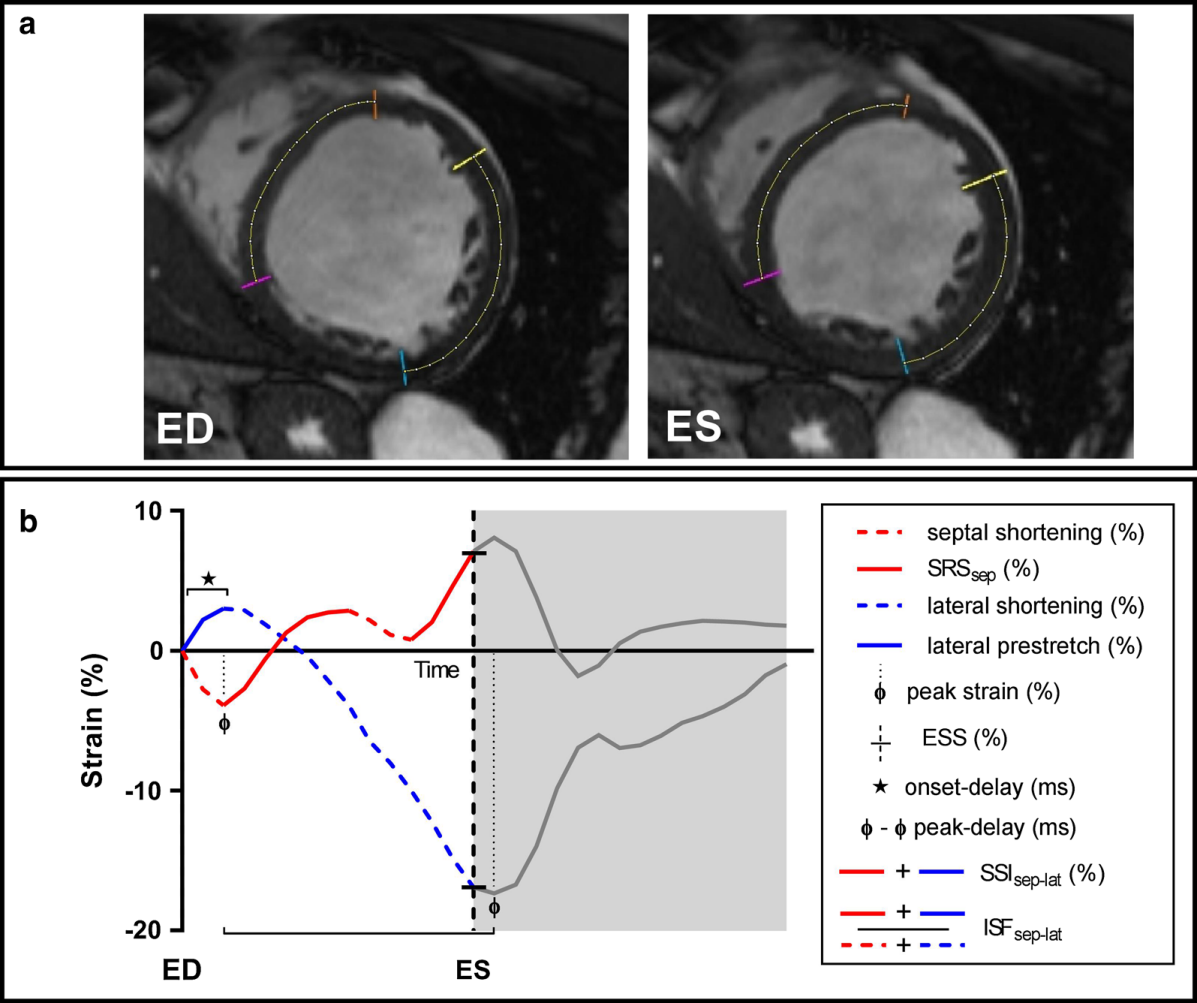
SLICE analysis and measurement of strain parameters. **a** Typical example of a left bundle branch block (LBBB) patient with segment length in cine (SLICE) analysis of the septal and lateral wall in the end-diastolic (ED) and end-systolic (ES) frame. **b** Strain curves of the septal (red) and lateral (blue) wall with measurement of basic strain values as the maximal negative strain value (peak strain) and end-systolic strain value (ESS) at aortic valve closure (determined by cine imaging of the left ventricle (LV) in the three-chamber view); timing differences measured as the septal to lateral delay in onset contraction (onset-delay) and peak negative strain delay (peak-delay); and inefficient strain patterns measured as systolic rebound stretch of the septum (SRS_sep_), systolic stretch index (SSI_sep-lat_) and internal stretch index (ISF_sep-lat_)

A detailed description of the SLICE analysis post-processing steps has been published previously [9]. In brief, the mid-LV slice position with short-axis cine images was selected (QMassMR version 7.6, Medis). Two endocardial anatomic landmarks (trabeculae) near the right ventricular (RV) insertion points, delimiting the septal segment, were chosen in the end-diastolic (ED) frame. Marks were placed perpendicular to the myocardium following the anatomic landmarks throughout all phases. This was repeated for the lateral wall segment. Localization of lateral wall landmarks was performed by drawing a straight line from the RV insertion points through the LV center point to the opposite site (see Additional file 1: Figure S1). Subsequently, marked cine images were exported to *ImageJ* to measure segment length measurements between the marks over the myocardium midline in each phase using the segmented line tool, see Fig. 1a. Segments lengths were expressed as a percentage of the ED segment length, and the frame-to-frame segment length change was plotted as a strain curve, see Fig. 1b.

### Strain parameters

Four subsets of strain parameters were evaluated. First, basic strain values were quantified by the septal and lateral peak negative strain (peak strain); and end-systolic strain (ESS) at the time of AVC (determined by cine imaging of the LV in the three-chamber view). Secondly, timing differences were measured by the septal-to-lateral delay in onset of shortening (onset-delay); and the septal-to-lateral time difference in peak contraction (peak-delay); Thirdly, strain pattern parameters included systolic rebound stretch of the septum (SRS_sep_); the systolic stretch index (SSI_sep-lat_); and the internal stretch index (ISF_sep-lat_). Lastly, septal strain patterns were visually classified to the following pre-specified patterns: double peaked systolic shortening (LBBB-1); early pre-ejection shortening followed by prominent systolic stretch (LBBB-2); and pseudonormal shortening with a late-systolic shortening peak and less pronounced end-systolic stretch (LBBB-3).

### Electrocardiographic and echocardiographic parameters

A detailed description of the ECG- and echocardiographic analysis has been published previously [10]. In brief, a baseline 12-lead ECG was recorded for QRS duration measurements and QRS morphology assessment. Subsequently, a 3D vector loop was constructed from the 12-lead ECG to calculate the QRS_AREA_. The presence of apical rocking was assessed during baseline echocardiography, and defined as a short systolic septal-to-lateral rocking motion of the apex [13]. In addition, the interventricular mechanical delay (IVMD) was measured as the timing difference between LV and RV pre-ejection intervals. Subsequently, the CRT-Age-Vectorcardiographic QRS_AREA_ -IVMD-Apical Rocking (CAVIAR) score was calculated as previously described in the MARC main study [10].

### Statistical analysis

Statistical analysis was performed in the study core lab (University Medical Center Groningen, Groningen, The Netherlands) by a specialized team led by a bioinformatician (BG) using the R software (R Foundation for Statistical Computing, Vienna, Austria). Continuous variables are expressed as mean ± standard deviation or in absence of a normal distribution as median and interquartile range. Categorical variables are presented as numbers and percentages. Strain parameters were compared between CRT responder and non-responder groups by an independent student t-test, or a non-parametric test when appropriate. Correlations between strain parameters and echocardiographic CRT response were assessed using the Pearson’s correlation coefficient or when normal distribution was absent, the Spearman’s Rho correlation coefficient. Receiver operating characteristics (ROC) curve analysis was used to find optimal cut-off values and determine predictive value of strain parameters. Univariable linear regression analysis was performed to assess the association of other (clinical, CMR and CAVIAR) parameters with echocardiographic CRT response. To test the additional value of SLICE strain analysis on top of conventional determinants of CRT response, multivariable linear regression analysis was performed by entering the best performing strain parameter (based on R) to a model with standard CMR parameters (model 1), clinical parameters (model 2) and the CAVIAR score parameters (model 3). Multiple testing was corrected for when appropriate. A *p*-value of < 0.05 was considered statistically significant.

## Results

After screening 63 patients, three patients were excluded from the analysis due to incomplete- or insufficient CMR image quality. Of the remaining 60 patients, 57 patients completed the 1-year follow up. One patient was lost to follow-up because of non-cardiac death (lung carcinoma), one withdrew informed consent, and one lacked sufficient image-quality during echocardiographic examination. Patient characteristics of the remaining 57 patients are presented in Table 1. Mean LVESV change after one year was − 32 ± 27% with 14 (25%) patients showing less than 15% LVESV reduction (CRT non-responders).

**Table 1:** Patient characteristics at baseline and twelve months follow-up

Variable	Total group (n = 57)	Responders (n = 43)	Non-responders (n = 14)	*P*-value
Age (years)	65 ± 10	63 ± 10	71 ± 9	0.013
Gender (n, % male)	30 (53%)	20 (47%)	10 (71%)	0.132
Etiology (n, % ICMP)	13 (23%)	5 (12%)	8 (57%)	0.001
ECG–QRS width (ms)	176 (165–188)	180 (168–194)	169 (152–176)	0.011
ECG–QRS morph (n, % LBBB)	45 (79%)	38 (88%)	7 (50%)	0.005
ECG–QRS area (µVs)	138 (118–159)	143 (126–168)	115 (78–126)	0.002
Echo–IVMD (ms)	50 ± 29	58 ± 25	24 ± 24	< 0.001
Echo–apical rocking (n, %)	39 (68%)	34 (79%)	5 (36%)	0.006
CAVIAR score (points)	3 ± 3	4 ± 2	0 ± 2	< 0.001
CMR–LVEDV (ml)	283 (227—338)	297 (231–380)	256 (220–297)	0.181
CMR–LVEF (%)	28 ± 10	27 ± 10	30 ± 6	0.092
CMR–Scar size (% LV mass)	1.3 (0.0–5.7)	0.0 (0.0–2.7)	5.7 (0.0–15.1)	0.028
Echo–Change in LVESV after 12 months (%)	− 32 ± 27	− 44 ± 18	4 ± 15	< 0.001
ECG–Change in QRS width after CRT (ms)	− 32 ± 28	− 37 ± 26	− 15 ± 29	0.017

Continuous variables are expressed as mean ± standard deviation or in absence of a normal distribution as median and interquartile range. Categorical variables are presented as numbers and percentages. *CAVIAR score* CRT-Age-Vectorcardiographic QRS_AREA_ -IVMD-Apical Rocking score, *ICMP* ischemic cardiomyopathy, *6MWT* 6 minute walk test, *LBBB* left bundle branch block, *IVMD* interventricular mechanical delay, *LVEDV* left ventricular end-diastolic volume, *LVEF* left ventricular ejection fraction, *CMR* cardiovascular magnetic resonance, *Echo* echocardiography, *ECG* electrocardiogram

### SLICE parameters and CRT response

**Fig. 2:**
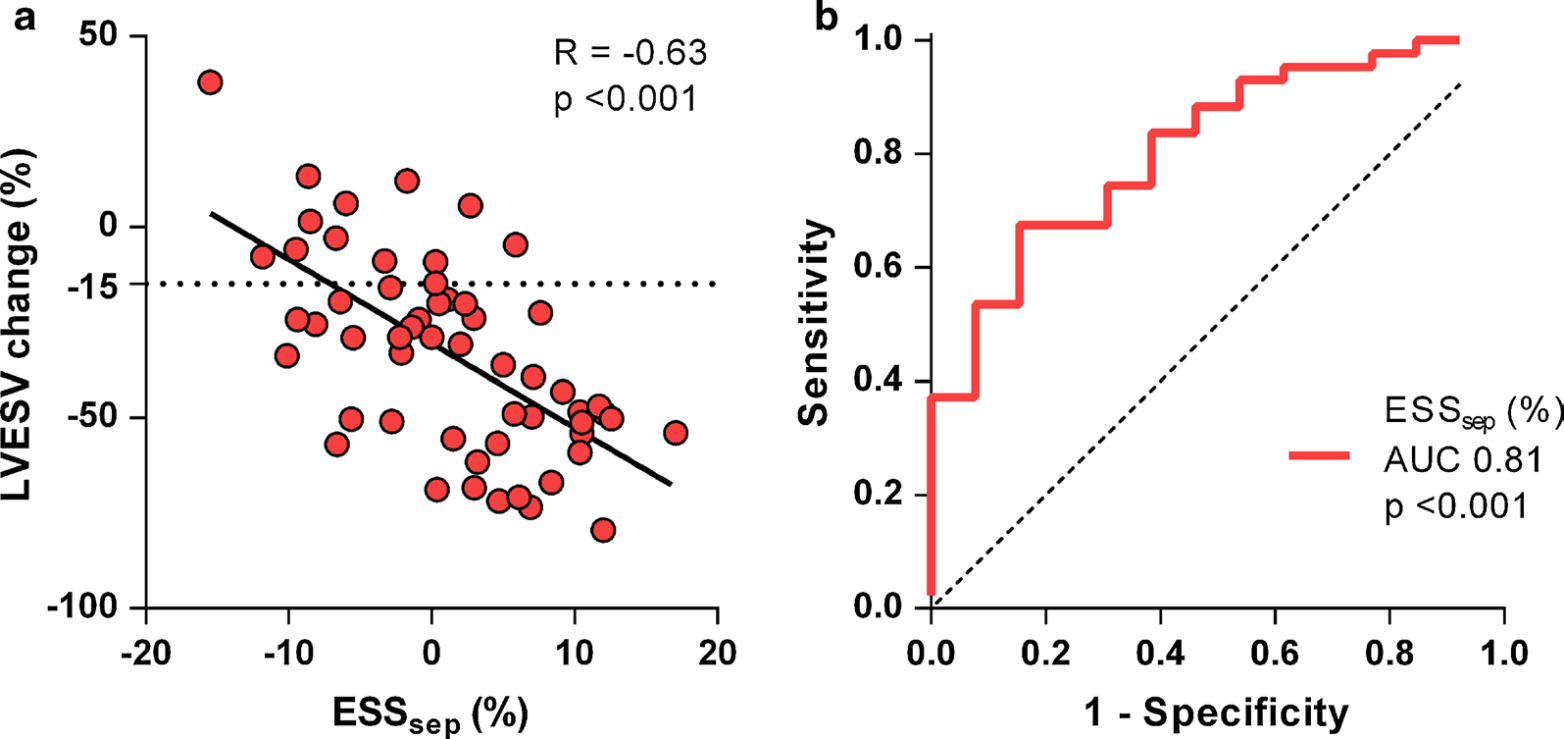
End-systolic septal strain and reverse remodeling after cardiac resynchronization therapy (CRT). **a** The end-systolic septal strain (ESS_sep_) parameter at baseline is strongly related with LV end-systolic volume (LVESV) change after CRT implantation. **b** ESS_sep_ shows good predictive value for CRT response (≥ 15% LVESV reduction) as demonstrated by receiver operating characteristic (ROC) curve analysis

**Fig. 3:**
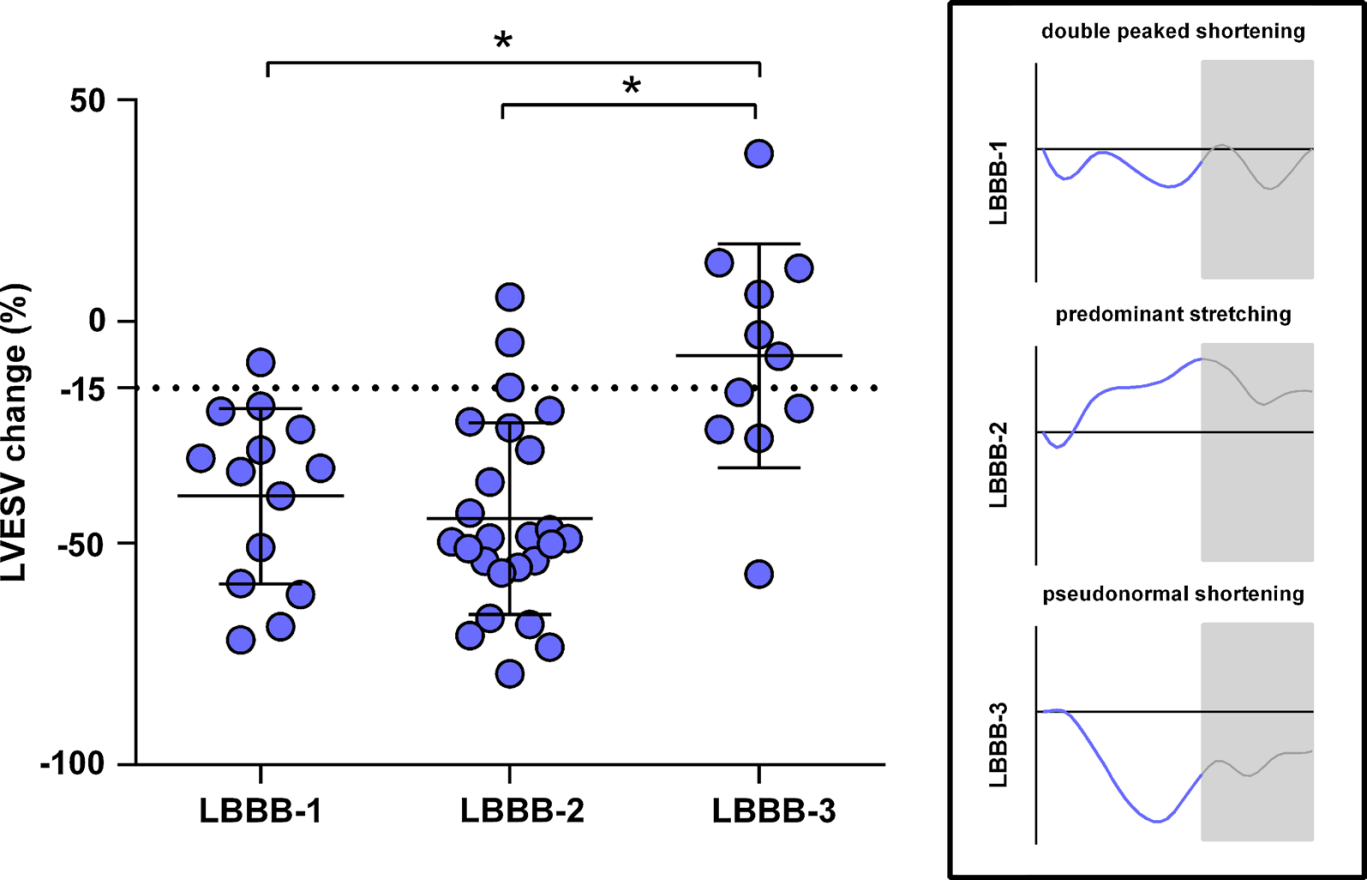
Visual classification of septal strain patterns to estimate CRT response. Septal strain patterns are classified to pre-specified categories: double peaked shortening (LBBB-1), predominant stretching (LBBB-2) or pseudonormal shortening (LBBB-3). Statistical differences between LBBB categories are marked with an asterisk

Strain values for the total patient group, and both responder and non-responder subgroups are presented in Additional file 1: Table S1 of the appendix. Basic strain values measured as peak strain in the septal and lateral wall region showed weak correlations with LVESV change as demonstrated in Table 2. In contrast, the basic strain parameter end-systolic septal strain (ESS_sep_) showed the highest correlation coefficient of all parameters (R − 0.63, *p* < 0.001) with good predictive value for CRT response (AUC 0.81, *p* < 0.001, Fig. 2) and an optimal cut-off of 0.3%. Comparing subgroups by QRS morphology, correlation of ESS_sep_ with CRT response was stronger within interventricular conduction delay (IVCD) patients (R − 0.87, *p* < 0.001) compared with LBBB patients (R − 0.48, *p* = 0.001). Timing parameters onset-delay and peak-delay were unrelated to LVESV change and yielded no predictive value for CRT response (Additional file 1: Table S2). On the other hand, strain pattern parameters all correlated with LVESV change (SRS_sep_ R − 0.56; SSI_sep-lat_ R − 0.53; ISF_sep-lat_; R − 0.58, all *p* < 0.001). As demonstrated in Fig. 3, septal strain curves were visually classified as LBBB-1 pattern in 25%, LBBB-2 in 44%, and LBBB-3 in 32% of the patients. Patients with pattern LBBB-1 or LBBB-2 demonstrated significantly more reverse remodeling compared to pattern LBBB-3. In addition, the response rate in the LBBB-1 and LBBB-2 was a two-fold higher than the response rate in the LBBB-3 group (88% / 93% versus 44%), see Additional file 1: Table S3. Comparing electrical parameters between the LBBB groups we found QRS morphology to be more frequently IVCD in LBBB-3 (44%) compared to LBBB-1 (29%) or LBBB-2 (0%) patients (p = 0.001 for comparison). However, no differences in QRS duration were found between groups.

**Table 2:** Correlation of SLICE-derived strain parameters with CRT response (left ventricular end-systolic volume (LVESV) change after 12 months)

	Variable	R	*p-*value
Basic strain parameters	Peak strain_sep_ (%)	− 0.37	0.004
	Peak strain_lat_ (%)	0.33	0.012
	ESS_sep_ (%)	− 0.63	< 0.001
	ESS_lat_ (%)	0.44	< 0.001
Timing parameters	Onset-delay (ms)	0.06	0.669
	Peak-delay (ms)	− 0.22	0.098
Strain pattern parameters	SRS_sep_ (%)	− 0.56	< 0.001
	SSI_sep-lat_ (%)	− 0.53	< 0.001
	ISF_sep-lat_	− 0.58	< 0.001

*R* correlation coefficient with LVESV change after twelve months; *Peak strain* peak negative strain; *ESS* end-systolic strain; *onset-delay* septal to lateral delay in onset contraction; *peak-delay* septal to lateral time difference in peak shortening; *SRS*_*sep*_ systolic rebound stretch of the septum; *SSI*_*sep-lat*_ systolic stretch index; *ISF*_*sep-lat*_ internal stretch factor

### Clinical parameters and CRT response

CRT responders were younger, had relatively less often ischemic etiology and wider QRS duration compared to non-responders (Table 1). LV lead position was lateral in 67%, posterolateral in 24% and anterolateral in 8% (left anterior oblique view) and basal in 20%, mid in 61% and apical in 18% (Right anterior oblizue view). LV lead location was congruent with scar location on LGE imaging in 18% of 47 patients. Patients with scar at LV lead location showed less LVESV reduction compared to others (− 13 ± 22% vs. − 36 ± 24%; p = 0.008). The LV capture threshold was 1.2 ± 0.7 V and was not related to presence of scar. Standard CMR parameters that correlated with reverse remodeling after CRT in univariable linear regression analysis included LV end-diastolic volume (LVEDV), LVEF, scar size and scar at LV lead location as demonstrated in Table 3. Addition of the best performing strain parameter, ESS_sep_, to standard CMR parameters in a multivariable model (model 1) showed ESS_sep_ to be independently related to LVESV change whereas other CMR parameters were not, although there was a trend towards significance for scar size. Clinical parameters that were associated with CRT response included age, gender, etiology, QRS duration and QRS morphology. In a multivariable model with clinical determinants (model 2), ESS_sep_ showed to be independently related to LVESV change together with age. The CAVIAR score parameters age, QRS_AREA_, IVMD, and apical rocking, were all significantly associated with CRT response. Addition of ESS_sep_ to CAVIAR (model 3), showed ESS_sep_ to be independently related to LVESV change together with age and QRS_AREA_.

**Table 3:** Linear regression analysis

	Univariable analysis			Model 1		
CMR parameters / ESS_sep_	Beta	95% CI	*P*-value	Beta	95% CI	*P*-value
CMR–LVEDV (per 10 ml)	− 0.80	− 1.53 to − 0.07	0.028	0.03	− 0.86–0.93	0.938
CMR–LVEF (per %)	0.79	0.08–1.51	0.029	0.42	− 0.41–1.24	0.312
CMR–Scar size (per % LV mass)	2.22	1.21–3.23	< 0.001	1.32	− 0.20–2.83	0.088
Scar at LV lead location (yes)	12.54	3.81–21.27	0.006	− 4.07	− 25.0–16.9	0.696
CMR–ESS_sep_ (per %)	− 2.20	− 2.94 to − 1.47	< 0.001	− 1.25	− 2.29 to − 0.21	0.019

Clinical parameters / ESS_sep_				Model 2		
Age (per year)	1.06	0.43–1.70	0.001	0.68	0.17–1.20	0.009
Gender (male)	17.3	3.94–30.67	0.011	− 1.24	− 12.63–10.16	0.831
Etiology (ICMP)	30.89	16.19–45.58	< 0.001	12.20	− 1.68–26.08	0.085
ECG–QRS width (per ms)	− 0.29	− 0.55 to − 0.02	0.033	− 0.08	− 0.30–0.14	0.481
ECG–QRS morph (LBBB)	− 29.12	− 44.62 to − 13.63	< 0.001	− 9.58	− 23.80–4.63	0.186
CMR–ESS_sep_ (per %)	− 2.20	− 2.94 to − 1.47	< 0.001	− 1.32	− 2.18 to − 0.47	0.002

CAVIAR components / ESS_sep_				Model 3		
Age (per year)	1.06	0.43–1.70	0.001	0.63	0.14–1.13	0.013
ECG–QRS area (per μVs)	− 0.32	− 0.45 to − 0.19	< 0.001	− 0.18	− 0.30 to − 0.05	0.005
Echo–IVMD (per ms)	− 0.52	− 0.73 to − 0.31	< 0.001	0.12	− 0.35–0.10	0.288
Echo–apical rocking	− 26.08	− 39.59 to − 12.56	< 0.001	− 2.66	− 15.56–10.25	0.687
CMR–ESS_sep_ (per %)	− 2.20	− 2.94 to − 1.47	< 0.001	− 1.51	− 2.35 to − 0.67	< 0.001

*LVEDV* left ventricular end-diastolic volume, *LVESV* left ventricular end-systolic volume, *LVEF* left ventricular ejection fraction, *ESS*_*sep*_ end-systolic septal strain, *ICMP* ischemic cardiomyopathy, *CAVIAR score* CRT-Age-Vectorcardiographic QRS_AREA_ -IVMD-Apical Rocking score, *IVMD* interventricular mechanical delay, *LBBB-3* visual classification of strain pattern 3

## Discussion

This study is the first to demonstrate predictive value of the novel SLICE strain technique for functional LV recovery after CRT implantation. Various SLICE strain parameters showed to be closely related with CRT response after 1 year. When comparing different types of strain parameters, strain pattern- rather than timing variables correlated with CRT response. Of all strain parameters, end-systolic septal strain demonstrated the strongest correlation with LV reverse remodeling after 1 year. Moreover, multivariable regression analysis showed end-systolic septal strain to be additive to clinical (age) and electrical (QRS_AREA_) parameters in estimating potential benefit from CRT.

### Comparison of strain parameters

In patients with LBBB, slow cell-to-cell LV conduction results in a time-difference of electrical activation between the septal and lateral wall. Although SLICE analysis revealed mechanical contraction always to be delayed in the lateral wall relative to the septal wall, absolute time delays between the septal and lateral wall (onset-delay; peak-delay) were unrelated to the amount of echocardiographic response after CRT. These findings are in agreement with those of the predictors of response to cardiac resynchronization therapy (PROSPECT) trial, showing disappointing results of timing (i.e. dyssynchrony) parameters [14]. More recent studies suggest strain pattern- (i.e. discoordination) rather than timing parameters to be related to CRT response [3, 4, 6]. In contrast to timing parameters, strain pattern parameters incorporate regional function by measuring myocardial contraction and stretching in percentage strain units. In line with previous reports, strain pattern parameters (SRS_sep_; SSI_sep-lat_; ISF_sep-lat_) all correlated with reverse remodeling after CRT and accurately predicted response to CRT [3, 4, 6].

### Septal strain analysis

During LBBB, systolic stretching is most profound in early-activated (i.e. septal) segments whereas contractile function is increased in late-activated (i.e. lateral wall) regions, resulting in an imbalanced septal-to-lateral work load ratio [15–17]. CRT subsequently recruits myocardial work from the septum, leading to a homogenized work distribution and improving cardiac pump efficiency [16–19]. In the present study, we found end-systolic strain of the septum (ESS_sep_) to be strongest related with reverse remodeling after CRT as illustrated in Fig. 2. Previously, we performed a systematic comparison of strain parameters using multiple strain imaging techniques (CMR-TAG; CMR-FT; STE) and found ESS_sep_ to be the best performing strain parameter in the prediction of CRT outcome irrespective of imaging technique [20]. The present study adds to the accumulating evidence that discoordination of the septum forms the mechanical substrate for functional LV improvement during electrical resynchronization. ESS_sep_ reflects net septum length change throughout systole and varied widely between patients ranging between − 15.5% (shortening) and 17.1% (stretching). These large differences in strain can be easily detected by SLICE and require septal analysis in only two (end-diastolic and end-systolic) frames (Fig. 1a). Time duration of the specific SLICE-ESS_sep_ measurement was only 12 ± 2 min. Previously, our validation study showed high agreement of SLICE-ESS_sep_ with gold standard CMR-TAG (ICC: 0.76) with excellent intra-observer reproducibility (ICC: 0.94) and good inter-observer reproducibility (ICC: 0.86) [9]. Reproducibility of SLICE-ESS_sep_ was higher compared to other strain parameters, presumably because of the wide spread in strain values. ROC curve analysis in the present study revealed an optimal cut-off value to predict CRT response around zero (0.3) percent. Patients with septal stretching rather than shortening during systole (positive numbers indicate stretching) are highly inefficient at baseline and leave more room for improvement in contractile function after CRT. These patients showed a three-fold larger reduction in LVESV compared to patients with preserved septal contraction. Alternatively, septal behavior can be evaluated in a subjective manner by the visual classification of septal strain curves to a pre-described pattern. Patients with a typical LBBB (i.e. LBBB-1 or LBBB-2) pattern demonstrated larger reductions in LVESV compared to patients with a pseudo-normal (i.e. LBBB-3) which is in line with previous studies [5, 8, 21]. However, calculation of quantitative strain parameters may be preferable as they are less dependent on the observer’s interpretation compared to the subjective classification of septal strain patterns.

### The role of SLICE strain analysis in clinical practice

In clinical practice, the role of CMR is of interest as it offers accurate LVEF measurement combined with LGE imaging to guide LV lead placement in CRT candidates [22–26]. Leyva et al. demonstrated LV lead deployment away from scarred myocardium to result in better clinical outcome while pacing in scarred myocardium was associated with the worst outcome [27]. In the present study, the role of SLICE strain analysis was compared to other CMR parameters to determine its value on top of standard information provided by CMR examination (model 1). Multivariable modeling showed ESS_sep_ to be the only parameter independently related to LVESV change, although there was a trend towards significance for scar size. Scar size and septal discoordination were interrelated (R = − 0.51; p < 0.001) as they possibly share mutual information on LV contractility [5]. Recent animal experiments showed that decreasing LV contractility by creating myocardial infarctions resulted in less septal stretching [28]. Information on global scarring is therefore partially incorporated in ESS_sep_ with lower values indicating poor CRT outcome.

**Fig. 4:**
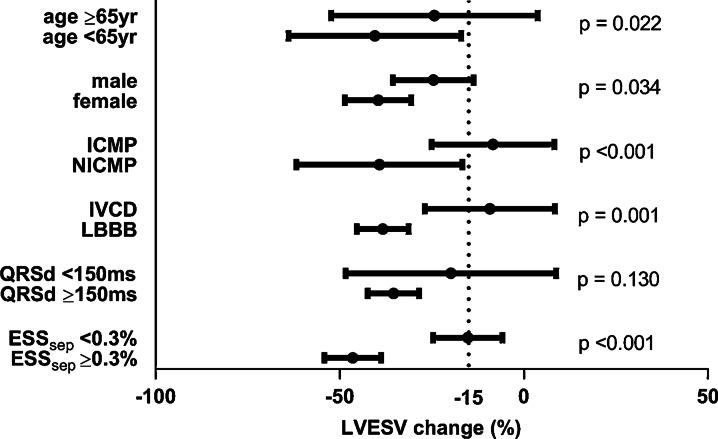
Clinical factors and ESS_sep_ influencing the likelihood of response to CRT. The magnitude of benefit from CRT is demonstrated for different subgroups divided by age; gender; etiology; QRS morphology; QRSd; and ESS_sep_. Compared to clinical factors, SLICE- ESS_sep_ demonstrates relatively large discriminative power

**Fig. 5:**
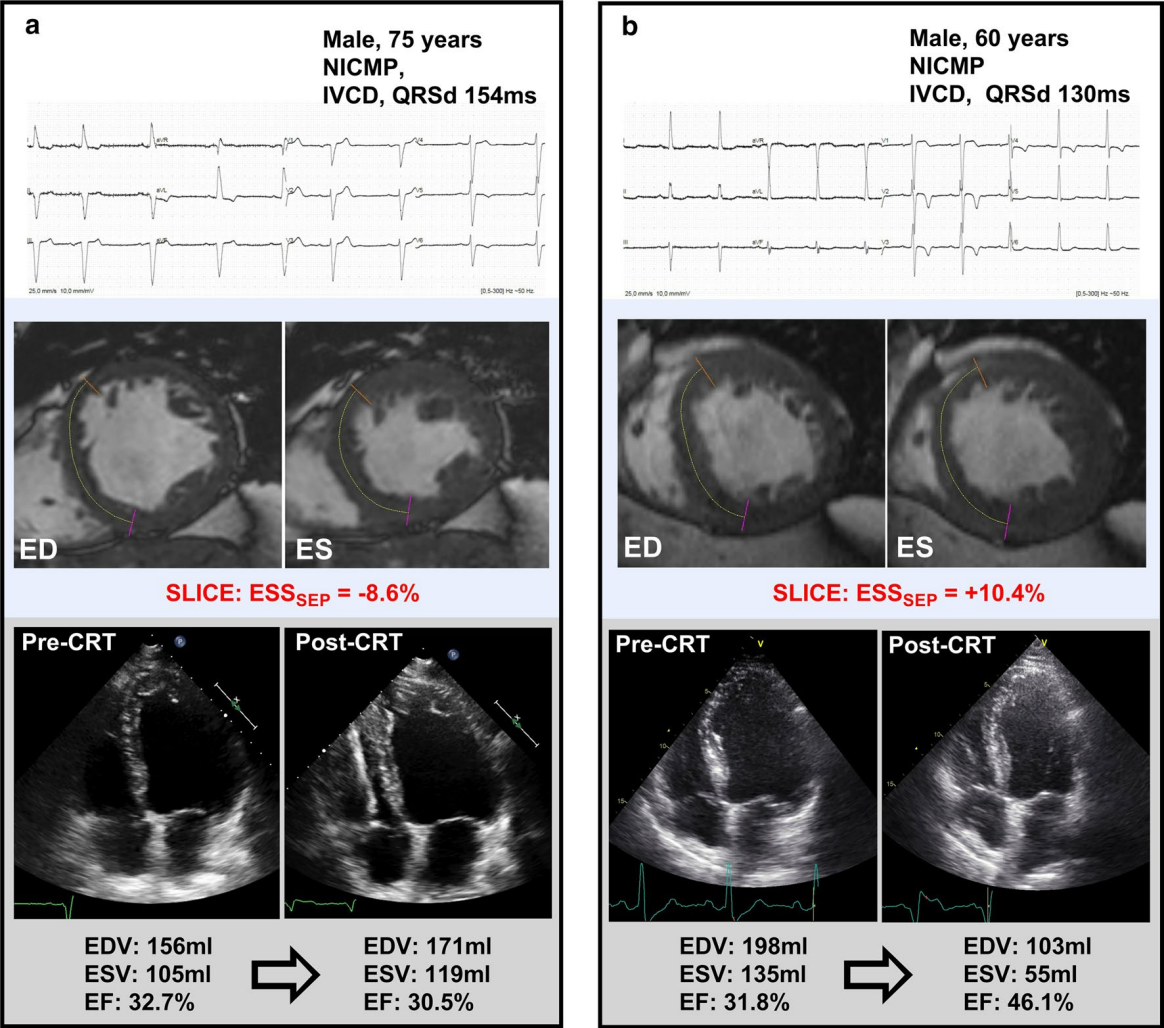
Two typical examples demonstrating the additional value of SLICE strain analysis in clinical practice. **a** Typical example of a grey-zone CRT candidate with mixed favorable (non-ischemic cardiomyopathy (NICMP); QRSd ≥ 150 ms), and unfavorable (male; age ≥ 65 years; IVCD) characteristics. SLICE analysis reveals a strongly negative ESS_sep_ and the patient shows no LV pump function improvement after CRT implantation. **b** Another patient with mixed favorable (NICMP; age < 65 years), and unfavorable (male; IVCD; QRSd < 150 ms) characteristics. This patient, however, demonstrates a strongly positive ESS_sep_ and becomes a CRT super-responder

Furthermore, the role of SLICE in relation to clinical parameters was explored. Clinical predictors of CRT response included age, gender, etiology, QRS width and QRS morphology (see Fig. 4), all considered to be traditional determinants of CRT response [1, 29]. The SLICE- ESS_sep_ parameter may add to the decision for CRT, especially in patients with IVCD in whom benefit from CRT is doubted. It can be appreciated in Fig. 4 that there is large variation in response among IVCD patients (ranging from non-response to super-response). Whereas strict LBBB is usually accompanied by septal discoordination (resulting in CRT response), this is much less certain in patients with IVCD. It could be hypothesized that electro-mechanical dissociation plays a larger role in IVCD than LBBB patients. Additional SLICE analysis may be used to confirm “true LBBB” septal discoordination in these patients. We found ESS_sep_ to provide predictive value over QRS morphology (model 2). Moreover, predictive performance of ESS_sep_ was stronger within IVCD patients than LBBB patients. The potential value of ESS_sep_ in IVCD patients is illustrated by two examples in Fig. 5. It should be noted, however, that IVCD patients composed of only 21% of the study population.

Lastly, the value of SLICE was evaluated with respect to a specially designed prediction model in the MARC main study (model 3). The CAVIAR response score incorporates age, QRS_AREA_ derived by vector-loop ECG analysis and two echocardiographic parameters being IVMD and detection of apical rocking [10]. Addition of ESS_sep_ to CAVIAR in multivariable analysis showed ESS_sep_ to be an independent predictor of response together with age and QRS_AREA_, whereas echocardiographic parameters (IVMD; apical rocking) were expelled from the model. These findings indicate that response to CRT is multifactorial [2], and that combining clinical information (age) together with electrocardiographic (QRS_AREA_) and mechanical information (SLICE-ESS_sep_) may improve patient selection for CRT. As QRS_AREA_ can be measured from a standard 12-lead ECG and SLICE-ESS_sep_ from standard CMR cine images, the work-up of CRT candidates is feasible in clinical practice.

### Limitations

Some limitations need to be recognized. First, this sub-study of the MARC involves a subset of patients who underwent additional CMR examination. Although patient characteristics were comparable with the overall MARC population, this may have introduced some selection bias. As a next step, predictive performance of SLICE-ESS_sep_ should be evaluated in a validation cohort which is part of future work. Secondly, 18% of patients had their LV lead implanted in a region with myocardial scarring. Targeting LV lead placement outside scar regions could potentially improve response to CRT and may impact predictive value of ESS_sep_ albeit not being investigated in the present study. Thirdly, CMR feature tracking (CMR-FT) post-processing software is now commercially available and offers automated strain analysis on standard CMR cine images. Nevertheless, CMR-FT has several downsides as it requires the purchase of commercial software and the user is not able to track and trace the analysis steps. SLICE, on the other hand, can be performed without the use of specialized software tools. Fourthly, strain parameters that require SLICE analysis of the entire strain curve may take a long processing time (up to 60 min). Measuring the specific ESS_sep_ parameter, however, requires only two SLICE measurements and can be performed in around twelve minutes. Furthermore, the SLICE analysis could be standardized by implementing radial taglines to standard cine imaging (Additional file 1: Figure S2). Lastly, SLICE relies on strain measures from in-plane motion of anatomic landmarks. However, the apparent in-plane movements may also be caused by through-plane displacements of oblique or tapering structures that form the anatomic landmarks. For this reason, we could only analyze the mid-LV slice, since this plane is relatively motion independent. Nevertheless, variation in strain values between basal, mid- and apical LV segments are relatively small [30].

## Conclusions

The practicable SLICE strain technique helps the clinician to estimate potential benefit from CRT by analyzing standard CMR cine images without the need for commercial software. Of all strain parameters, end-systolic septal strain (ESS_sep_) demonstrated the strongest correlation with reverse remodeling after CRT. This parameter can be measured in around 12 min and may be of special interest in patients with non-strict LBBB morphology in whom CRT benefit is doubted. New clinical trials are needed to determine whether detection of septal discoordination yields additive value to traditional CRT parameters in clinical practice.

## Supplementary Information

**Additional file 1: Table S1.** Comparison of strain parameters between CRT responders and non-responders. **Table S2.** Predictive value of strain parameters for CRT response (≥ 15% reduction in LVESV). **Table S3.** Septal strain patterns and CRT response. **Figure S1.** Localization of the anatomical landmarks. **Figure S2.** Modification of the SLICE technique by implementing radial taglines.

## Data Availability

The datasets generated and/or analysed during the current study are not publicly available due to planned future publications.

## Abbreviations

AV: Atrioventricular
AVC: Aortic valve closure
bSSFP: Balanced steady state free precession
CAVIAR: CRT-age-vectorcardiographic QRSarea-RVMD-apical rocking score
CMR: Cardiovascular magnetic resonance
CRT: Cardiac resynchronization therapy
ECG: Electrocardiogram
ED: End-diastole
ESS: End-systolic septal strain
FT: Feature tracking
ISF_sep-lat_: Internal stretch factor
IVCD: Intraventricular conduction delay
IVMD: Interventricular mechanical delay
LBBB: Left bundle branch block
LGE: Late gadolinium enhancement
LV: Left ventricle/left ventricular
LVEDV: Left ventricular end-diastolic volume
LVEF: Left ventricular ejection fraction
LVESV: Left ventricular end-systolic volume
MARC: Markers and Response to CRT study
RV: Right ventricle/right ventricular
SLICE: Segment length in cine
SRS: Systolic rebound stretch
SSI: Systolic stretch index
TAG: Tagging
TE: Echo time
TR: Repetition time
VV: Interventricular
