# Supplementary material for: Segment length in cine (SLICE) strain analysis: a practical approach to estimate potential benefit from cardiac resynchronization therapy
Source: J Cardiovasc Magn Reson. 2021 Jan 11;23:4. doi: 10.1186/s12968-020-00701-4 (PMC7798189; doi:10.1186/s12968-020-00701-4)

**Additional Material**

Segment Length in Cine (SLICE) Strain Analysis: a Practical Approach

to Estimate Potential Benefit from Cardiac Resynchronization Therapy

**Additional TABLES**

Table S1. Comparison of strain parameters between CRT responders and non-responders

|  | **Variable** | **Total group (n=57)** | **Responders (n=43)** | **Non-responders (n=14)** | ***p-*value** |
| --- | --- | --- | --- | --- | --- |
| *basic strain parameters* | Peak strain_sep_ (%) | -5.4 (-8.6 – -2.9) | -4.7 (-7.9 – -2.5) | -7.8 (-12.8 – -5.0) | p = 0.011 |
|  | Peak strain_lat_ (%) | -13.6 ± 4.8 | -14.5 ± 4.6 | -10.8 ± 4.4 | p = 0.013 |
|  | ESS_sep_ (%) | 1.3 ± 7.3 | 3.1 ± 6.7 | -4.8 ± 6.1 | p <0.001 |
|  | ESS_lat_ (%) | -11.8 ± 5.5 | -13.0 ± 5.1 | -8.4 ± 5.5 | p = 0.012 |
| *timing*  *parameters* | onset-delay (ms) | 73 ± 42 | 74 ± 38 | 70 ± 53 | p = 0.794 |
|  | peak-delay (ms) | 249 (127 – 348) | 268 (124 – 351) | 201 (139 – 285) | p = 0.889 |
| *strain pattern*  *parameters* | SRS_sep_ (%) | 7.0 (2.5 – 12.1) | 7.5 (5.2 – 13.0) | 2.0 (1.3 – 4.0) | p <0.001 |
|  | SSI_sep-lat_ (%) | 8.6 (5.1 – 14.8) | 12.1 (6.8 – 15.2) | 3.2 (2.0 – 7.5) | p <0.001 |
|  | ISF_sep-lat_ | 0.48 ± 0.29 | 0.56 ± 0.27 | 0.25 ± 0.19 | p <0.001 |
| *visual*  *classification* | septal pattern:  LBBB1  LBBB2  LBBB3 | 14 (25%)  25 (44%)  18 (32%) | 13 (30%)  22 (51%)  8 (19%) | 1 (7%)  3 (21%)  10 (71%) | p = 0.001 |

Peak strain, peak negative strain; ESS, end-systolic strain; onset-delay, septal to lateral delay in onset contraction; peak-delay, septal to lateral time difference in peak shortening; SRS_sep_, systolic rebound stretch of the septum; SSI_sep-lat_, systolic stretch index; ISF_sep-lat_, internal stretch factor; LBBB-1, double-peaked shortening; LBBB-2, initial shortening followed by predominant stretching; LBBB-3, pseudo-normal shortening.

Table S2. Predictive value of strain parameters for CRT response (≥15% reduction in LVESV)

|  | **Variable** | **AUC** | ***p-*value** | **cut-off value** | **sens/spec** |
| --- | --- | --- | --- | --- | --- |
| *basic strain parameters* | Peak strain_sep_ (%) | 0.73 | 0.004 | -3.5% | 44% / 100% |
|  | Peak strain_lat_ (%) | 0.70 | 0.007 | -12.5% | 67% / 71% |
|  | ESS_sep_ (%) | 0.81 | <0.001 | 0.3% | 67% / 85% |
|  | ESS_lat_ (%) | 0.73 | 0.006 | -9.6% | 70% / 64% |
| *timing*  *parameters* | onset-delay (ms) | 0.52 | 0.376 | 45ms | 81% / 43% |
|  | peak-delay (ms) | 0.51 | 0.438 | 264ms | 53% / 71% |
| *strain pattern*  *parameters* | SRS_sep_ (%) | 0.80 | <0.001 | 4.5% | 81% / 79% |
|  | SSI_sep-lat_ (%) | 0.82 | <0.001 | 5.9% | 79% / 71% |
|  | ISF_sep-lat_ | 0.82 | <0.001 | 0.30 | 79% / 79% |

AUC, area under the curve; sens/spec, sensitivity/specificity; for other abbreviations see table S1.

Table S3. Septal strain patterns and CRT response

| **N = 57** | **LVESV change (%)** | **Response rate (n, %)** |
| --- | --- | --- |
| LBBB-1 | -39 ± 20*  n=14 | 13 (93%) |
| LBBB-2 | -44 ± 22†  n=25 | 22 (88%) |
| LBBB-3 | -10 ± 25*†  n=18 | 8 (44%) |

LBBB-1 (double-peaked shortening) and LBBB-2 (predominant stretching) both represent typical LBBB strain patterns. In contrast, LBBB-3 (pseudo-normal shortening) represents non-typical LBBB pattern. Statistical differences between LBBB categories per imaging modality are marked with: * pattern 1 vs. 3, † pattern 2 vs. 3.

**Additional FIGURE LEGENDS**

Figure S1: *Localization of the anatomical landmarks*

Step-by-step illustration to define anatomical landmarks in the end-diastolic mid-LV short-axis cine. First, a straight line was drawn from the anterior RV insertion point through the LV center point to locate the posterolateral region (diagram A). Subsequently, marks were placed perpendicular to the myocardium at the nearest trabecula that were traceable throughout the cardiac cycle (diagram B). This procedure was repeated for the posterior RV insertion point and the anterolateral region (diagram C). Septal and lateral segment length was measured in *ImageJ* using a segmented line (diagram D).

Figure S2: *Modification of the SLICE technique by implementing radial taglines*

Two radially oriented taglines are placed at end-diastole to form the reference points for each segment throughout the cardiac cycle. In future studies, contour tracing of these cine images could enable LV function assessment combined with fully automated SLICE strain analysis.

**Additional figures**

Figure S1: *Localization of the anatomical landmarks*

**
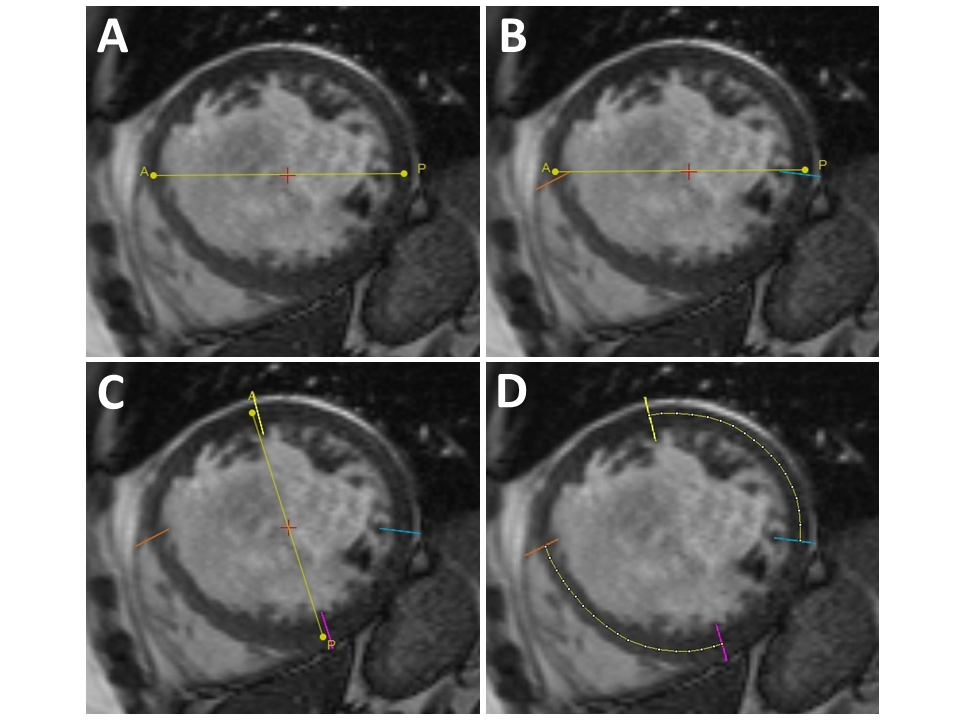
**

Figure S2: *Modification of the SLICE technique by implementing radial taglines*


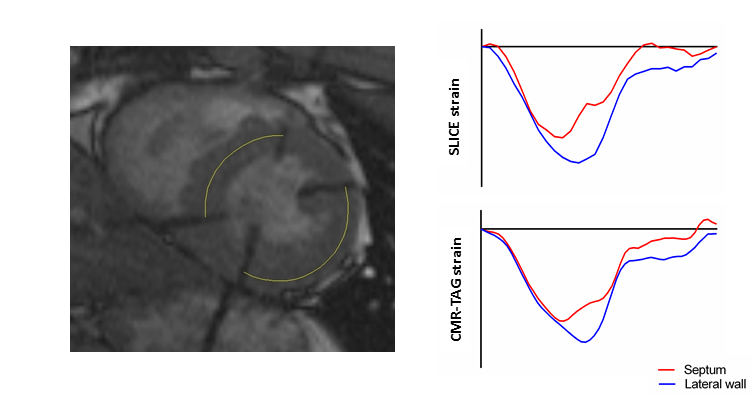

Supplement: Supplementary file 1 — Additional file 1: Table S1. Comparison of strain parameters between CRT responders and non-responders. Table S2. Predictive value of strain parameters for CRT response (≥ 15% reduction in LVESV). Table S3. Septal strain patterns and CRT response. Figure S1. Localization of the anatomical landmarks. Figure S2. Modification of the SLICE technique by implementing radial taglines. [file 12968_2020_701_MOESM1_ESM.docx]
